# Maternal Complications and Women's Behavior in Seeking Care from Skilled Providers in North Gondar, Ethiopia

**DOI:** 10.1371/journal.pone.0060171

**Published:** 2013-03-28

**Authors:** Abebaw Gebeyehu Worku, Alemayehu Worku Yalew, Mesganaw Fantahun Afework

**Affiliations:** 1 Department of Reproductive Health, Institute of Public Health, University of Gondar, Gondar, Ethiopia; 2 Department of Preventive Medicine, School of Public Health, Addis Ababa University, Addis Ababa, Ethiopia; 3 Department of Reproductive Health and Health Service Management, School of Public Health, Addis Ababa University, Addis Ababa, Ethiopia; Boston Children's Hospital, United States of America

## Abstract

**Background:**

Maternal complications are morbidities suffered during pregnancy through the postpartum period of 42 days. In Ethiopia, little is known about women's experience of complications and their care-seeking behavior. This study attempted to assess experiences related to obstetric complication and seeking assistance from a skilled provider among women who gave birth in the last 12 months preceding the study.

**Methods:**

This study was a cross-sectional survey of women who gave birth within one year preceding the study regardless of their delivery place. The study was carried out in six selected districts in North Gondar Zone, Amhara Region. Data was collected house-to-house in 12 selected clusters (kebeles) using a pretested Amharic questionnaire. During the survey, 1,668 women were interviewed. Data entry was done using Epi Info version 3.5.3 and was exported to SPSS for analysis. Logistic regression was applied to control confounders.

**Results:**

Out of the total sample, 476 women (28.5%, 95% CI: 26.4%, 30.7%) reported some kind of complication. The most common complications reported were; excessive bleeding and prolonged labor that occurred mostly at the time of delivery and postpartum period. Out of the total women who faced complications, 248 (52.1%, 95% CI: 47.6%, 56.6%) sought assistance from a skilled provider. Inability to judge the severity of morbidities, distance/transport problems, lack of money/cost considerations and use of traditional options at home were the major reasons for not seeking care from skilled providers. Belonging to a wealthier quintile, getting antenatal care from a skilled provider and agreement of a woman in planning for possible complications were significantly associated with seeking assistance from a skilled provider.

**Conclusion:**

Nearly half of the women who faced complications did not use skilled providers at the time of obstetric complications. Cognitive, geographic, economic and cultural barriers were involved in not using skilled maternal care.

## Introduction

Many mothers living in developing countries continue to die from pregnancy related morbidities each year [1], [2]. Assessments of progress towards the Millennium Development Goals (MDGs) have found that the least progress is made in MDG 5 (reducing maternal mortality by three-fourth), particularly in Sub-Saharan Africa [3], [4]. Ethiopia is one of the countries with the highest maternal mortality ratio which is currently estimated at 676 maternal deaths per 100,000 live births [5]. Maternal deaths are the tip of the iceberg. For every maternal death, about 20 more women are estimated to suffer from acute or chronic pregnancy related illnesses [6]. Worldwide, an estimated 10–20 million women develop physical or mental disabilities every year as a result of complications or their poor management [6].

Maternal morbidities include all illnesses and complications associated with child bearing short of death. Maternal morbidity takes many forms. It can be an acute or a chronic condition, which is either recognized or acknowledged as an illness or is not apparent to the respondent. The seriousness of a specific morbidity also varies; some are instantaneously lethal, while others are potentially lethal, disabling, or simply discomforting. Morbidity events may be single episodes which resolve spontaneously, are treated, or have permanent incurable effects, while others may recur occasionally or frequently [7]. Acute maternal morbidities are complications suffered during pregnancy through the postpartum period of 42 days that may directly cause maternal deaths [8]. These can be expressed in different terms according to their severity. Obstetric or maternal complications are acute conditions which include antepartum or postpartum hemorrhage, prolonged or obstructed labor, postpartum sepsis, complications of abortion, pre-eclampsia/eclampsia, ectopic pregnancy, and ruptured uterus [9]. Overall an estimated 15% of women are expected to experience maternal complications [10]. Women without pregnancy complications can also rapidly develop such problems and may need timely access to life saving services [11]. As observed in many studies, severe maternal complications will have potentially devastating consequences on women and their families, and recovery can be slow, with lasting sequelae [12], [13].

Since the majority of the complications are unpredictable, an appropriate use of skilled birth attendance and supportive emergency obstetric care are critical interventions for the survival of the mother. Maternal death can shortly follow if there are no such services. The estimated average interval between the onset of major obstetric complications and death, in the absence of medical interventions, is two hours for postpartum hemorrhage, twelve hours for antepartum hemorrhage, one day for a ruptured uterus, two days for eclampsia, three days for prolonged labor and six days for infection (puerperal sepsis) [10].

In Ethiopia, skilled maternal service utilization is very low. The recent (2011) Ethiopian demographic and health survey report showed that only 34%, 10% and 6% of women have Antenatal care (ANC), delivery and postnatal care by a skilled provider, respectively [5]. This indicated that many mothers with complications did not get a timely referral to emergency obstetric care facilities. However, there are limited evidences related to the proportion of women affected by maternal complications and seek care for the problems. Moreover, detailed evidences related to the reasons for not seeking care at the time of complication were lacking in the study area. This study focused on understanding such reasons based on women's perceptions and beliefs towards the risks as well as their experiences on obstetric complications and care-seeking behavior, which are imperative for planning appropriate interventions. Accordingly, the purpose of this study was to assess women's experiences of obstetric complications and their behavior in seeking assistance from skilled providers among women who gave birth in the last 12 months prior to the study in North Gondar, Ethiopia.

## Methods

This study was a cross-sectional survey of women (age of 15 years and above) who gave birth within one year preceding the study regardless of their delivery place. The study was conducted over three months period (January–March 2012) in six districts of North Gondar Zone, Amhara Region. Gondar is located 740 kilometer northwest of Addis Ababa. At the time of the study, there were over three million inhabitants in North Gondar Zone, about 84.1% of which were rural dwellers. In the zone, there is only one referral hospital with a comprehensive emergency obstetric care. There are also about a hundred health centers and two rural hospitals. However, only the two rural hospitals and a few health centers fulfill signal functions for basic emergency obstetric care services. At the time of complications, clients need to travel long distances to get functional emergency obstetric facilities. North Gondar, which is the largest zone of the region, has a difficult topography. It is essential to understand the difficulties faced in seeking health care, especially at the time of obstetric complications in this area. Women who had births within the past one-year preceding the survey were participants of the study. The study was part of big study dealing with maternal service utilization and determinants of use in North Gondar Zone. The sample size was estimated based on a single population proportion formula. By assuming a 5% margin of error, 95% confidence level, and at least 50% of women expected to seek assistance from a skilled provider, about 384 women with complications were needed to estimate the rate of skilled maternal care for complications.

Multistage sampling procedure was applied to select study participants. Initially, six districts of North Gondar were selected. Then twelve “kebeles” (two kebeles per district) were selected randomly. The “kebeles” (used as clusters) are the lowest administrative units in Ethiopia. All eligible women living in the selected clusters were studied. The data collection tool was adapted from DHS and other maternal and child health surveys. The questionnaire included a range of closed and open-ended questions on socio-demographic characteristics, awareness, perceptions and experiences related to maternal complications and use of skilled maternal care services. Questions related to care of complications include: the type of provider who gave assistance (if assistance was sought); the type of facility; person who made the decision for care-seeking; the type of transport used; estimated time and distance to reach facility; waiting time at health facility; the major type of care provided at a health facility, and the cost expenditure for transport and care. The data collectors and supervisors were diploma and degree graduates, respectively. A total of 36 data collectors and supervisors (2 data collectors and 1 supervisor for each kebele) were trained and deployed. The interviews were conducted in Amharic after questions were pretested for cultural appropriateness and clarity.

In the context of this study, skilled maternal care refers to maternity services by a health professional with midwifery skills that can be provided at different levels (home, health centers or hospitals). Health professionals, who have been effectively educated and trained in the skills needed to manage normal (uncomplicated) pregnancies, childbirth and the immediate postnatal period, as well as in the identification, management or referral of complications are categorized as skilled care providers [14], [15]. In the study, skilled providers include midwives, nurses, health officers and doctors while non-skilled providers include health extension workers (HEWs), traditional birth attendants (TBAs) and relatives or family members who cannot fulfill the definition of a skilled provider.

The unprompted responses (listed responses spontaneously for open questions) and prompted responses (selected responses from the available choices) were reviewed. Then data cleaning was performed to check for accuracy, consistency, missed values and variables. Data entry was done using Epi Info version 3.5.3 and was exported to SPSS for analysis. Tables, graphs and texts were used to present the study findings. To indicate the accuracy of the estimates, 95% CI was constructed for proportions. Logistic regression was applied to control confounding variables.

### 

#### Ethics statement

Before the commencement of the study, ethical clearance was obtained from the Institutional Review Board of the College of Health Sciences, Addis Ababa University. During data collection, consent was sought and all participants signed written agreements (consent forms) after they were introduced to the purpose of the study and informed about their rights to interrupt the interview on desire. To ensure confidentiality, names were avoided in reporting the results of the study. In addition, the collected information was locked with a key (hard copies) and by passwords (soft copies). The Institutional Review Board approved all ethical procedures, based on awareness about the type of study (that it was harmless to study subjects) and the characteristics of the participants (women aged 15 years and above who had given birth within one year preceding the survey.

## Results

### Socio-demographic characteristics of the study population

In the study, 1,668 eligible women were identified. The large majority (92.7%) of the women were married. At the time of their last birth, about 73.3% were between 20–34 years of age; 5.5% were teenagers (less than 20 years), and 21.2% were elderly gravid (35 years and above). The mean (±SD) age was 28.2 (±6.6) years, with a range of 16–51 years. The last birth for one-fifth of the mothers was the first, while it was the second or third for 39%; fourth or fifth for 26%, and more than fifth for 14%. The majority of the interviewed mothers (71%) and their husbands (63.4%) had no formal education (Table 1).

**Table 1:** Frequency and percentage distribution of respondents according to selected socio-demographic characteristics, North Gondar, 2012.

Characteristics		n	%
Residence (District)	Dabat	293	17.6
	Debark	244	14.6
	Dembia	294	17.6
	L.Armachiho	228	13.7
	Metema	306	18.3
	W.Belesa	303	18.2
Religion	Orthodox	1546	93.7
	Muslim	104	6.3
Ethnicity	Amhara	1536	92.7
	Qimant	78	4.7
	Tigrawi	43	2.6
Marital status	Never married	77	4.6
	Married	1537	92.7
	Divorced	37	2.2
	Widowed	7	0.4
Birth order	1	335	20.1
	2–3	657	39.4
	4–5	439	26.3
	6+	237	14.2
Mather's education	No education	1183	70.9
	Primary	327	19.6
	Secondary	138	8.3
	>Secondary	20	1.2
Husband's education	No education	1057	63.4
	Primary	430	25.8
	Secondary	154	9.2
	>Secondary	27	1.6
*Wealth index	Lowest	331	19.8
	Second	340	20.4
	Middle	306	18.3
	Fourth	357	21.4
	Highest	334	20.0

* The index of wealth status was computed by principal component analysis from ten variables (presence of own farmland, own toilet facility, bank account, mobile phone, electricity, roof of house with corrugated iron sheet, number of cows/oxen, horses/mules/donkeys, goats/sheep and chicken). The index was developed by categorizing sum of components in to five groups and it was ranked from poorest to wealthiest quintile.

### Awareness and perceptions related to obstetric complications and skilled maternal care

About 60% of women had heard about skilled maternal care (at the time of pregnancy, delivery and postpartum), and 59% had heard about the importance of birth preparedness (Table 2). Of the total unprompted responses, the main sources of information about the importance of birth preparedness were health professionals (24.9%), TBAs and HEWs (22.9%), and families (16.3%) which were mentioned by 44.6%, 41%, and 29.1% of the women, respectively.

**Table 2:** Awareness and perceptions related to obstetric complications and the need of skilled maternal care among women having birth within one year preceding the survey, North Gondar, 2012.

	From total sample	Sought assistance during complication (n = 476)		
Awareness and perceptions	Yes *n (%)	No(228) n (%)	Yes (248) n (%)	OR(95%CI)
Heard about skilled care (n = 1668)	995 (59.7)	135 (46.6)	155 (53.4)	1.15 (0.80, 1.66)
Heard about birth preparedness (n = 1668)	985 (59.1)	126 (45.0)	154 (55.0)	1.33 (0.92,1.91)
Respondent's known place to get skilled attendants (unprompted responses) (n = 1535)		175 (44.4)	219 (55.6)	2.29 (1.40, 3.75)
Government hospital	421 (27.4)			
Government health center	848 (55.2)			
Health post	48 (3.1)			
Private clinic	10 (0.7)			
Thought that some group of women are at higher risk of complications than others (n = 1579)	1146 (72.6)	139 (41.2)	198 (58.8)	2.536 (1.69, 3.82)
Group of women considered at higher risk by respondents (prompted responses)				
Advanced age (n = 1508)	1023 (67.8)			
Frequent births (n = 1505)	801 (53.2)			
Poor women (n = 1497)	763 (51.0)			
First pregnancy (n = 1508)	729 (48.3)			
Early age (n = 1505)	660 (43.9)			
Short stature (n = 1494)	653 (43.7)			
Multiple births (>5 five birth) (n = 1505)	621(41.3)			
Thought that the selected delivery attendants are better (safer) than the others (n = 1667)				
Health professionals	955 (57.3)	121 (44.5)	151 (55.5)	1.38 (0.96, 1.98)
TBA	575 (34.5)			
Relatives	97 (5.8)			
Agreed on the need of post natal check up (n = 1643)	901 (54.8)	113 (43.8)	145 (56.2)	1.43 (1.00, 2.06)
Agreed on the idea that leaving the house/compound/in the first seven days after delivery is safe (n = 1634)	248 (15.2)	26 (34.2)	50 (65.8)	1.96 (1.18, 3.28)
Agreed on the importance of planning delivery place (n = 1632)	1236 (75.7)	161 (44.5)	201 (55.5)	1.78 (1.16, 2.73)
**Agreed on the need of having a plan on possible complications (n = 1606)	1195 (75.5)	152 (42.1)	209 (57.9)	2.68 (1.73, 4.16)

* n represents those who say “Yes” and its percentage is calculated from total (Yes+No).

** Evaluation of agreement was considered based on the following question. “A woman should plan ahead of time what she will do if she has a serious health problem related to pregnancy or childbirth. Do you agree by this idea?”

In order to assess awareness of complications during pregnancy, childbirth, or soon after delivery, women were asked to list danger signs spontaneously. Out of the total unprompted responses, excessive bleeding (they called it ‘serakian’) (22%), prolonged labor (18.2%), and retained placenta (16.9%) were the top three listed danger signs. Such complications were mentioned by 65.3%, 54.2%, and 50.3% of women, respectively. The majority (55.2%) of the respondents mentioned health center as their palace to get a skilled attendant, while the rest 27.4%, 3.1%, and 0.7% mentioned government hospital, health post, and private clinic, respectively (Table 2).

Mothers were asked about their perceptions on complications and the need for skilled providers. About 72.6% thought that some groups of women were at higher risk of complications compared with others. Based on prompted responses, a significant proportion of the participants believed that women in advanced age (67.8%), women giving frequent births (53.2%), poor women (51%), women in their first pregnancy (48.3%), those with pregnancy in early age (43.9%), short women (43.7%), and women giving multiple births (41.3%) are at higher risk of developing complications. Women were also asked to mention the better (safer) delivery attendant according to their perception. About 57% perceived that health professionals were better and safer birth attendants, while 34.5% and 5.8% preferred TBAs and relatives, respectively (Table 2).

About 54.8% of the women agreed on the need for a postnatal check up. However, only a few (15.2%) of the respondents supported the idea that leaving home/compound/in the first seven days after delivery is safe (Table 2). Many of the respondents (72%) preferred to stay at home in the first 10 days. Most women, 75.7% and 75.5% agreed on the importance of planning the place of delivery and for possible complications, respectively (Table 2). In the bivariate analysis, the awareness of women about where to get skilled providers and the risk of pregnancy showed that there was a significant association with seeking skilled provider during complications. Moreover, agreement on postnatal check up, planning delivery place, planning for possible complication and belief that leaving compound in the postpartum period is safe showed a significant contribution to seek assistance at the time of complications (Table 2).

### Experiences of complications and assistance by a skilled provider

In total, 476 women (28.5%, 95% CI; 26.4%, 30.7%) reported some kind of complication. Ninety (5.4%; 95% CI, 4.3%, 6.5%), 224 (13.4%, 95% CI; 11.8%, 15.1%) and 274 (16.4%; 95% CI, 14.6%, 18.2%) women faced complications during pregnancy, intra-partum and postpartum periods. The most common reported complications were excessive bleeding (58.4%), and prolonged labor (23.7%). Most women encountered these complications at the time of delivery and immediate postpartum period (Table 3).

**Table 3:** Frequency and percentage distribution of self- reported complication encountered by women in their last birth within one year preceding the survey, North Gondar, 2012.

Type of self reported complications	Frequency and percentage by different time periods - n (%)			
	During pregnancy	During Intra-partum period	During Postpartum period	At least one complication throughout the periods
Excessive bleeding	72(80.0)	85(37.9)	221(80.7)	278(58.4)
Prolonged labor		113(50.4)		113(23.7)
Convulsion	6(6.7)	18(8.0)	10(3.6)	34(7.1)
High Fever/sepsis	5(5.6)		21(7.7)	21(4.4)
Body swelling	6(6.7)	6(2.7)	15(5.5)	20(4.2)
**Others	1 (1.1)	2(0.9)	7(2.6)	10 (2.1)
Total	90(100)	224(100)	274(100)	*476(100)

* Some complications can be persisted or repeated at different periods (pregnancy to postpartum) and the total values can be different from the summations.

** Stillbirth, fistula and severe anemia.

Out of the total women who faced complications, 282 (59.2%, 95% CI, 54.8%, 63.7%) sought assistance. Most, 248 (52.1%), of the assistances were by skilled providers (doctors (5.9%), health officers (4.4%) and nurses/midwives (39.5%)), and some, 34 (7.1%), were by non-skilled providers (Table 4).

**Table 4:** Type of providers assisting mothers at the time of complication in their last birth within one year preceding the survey, North Gondar 2012, n = 476.

			95% CI (%)	
		No (%)	Lower	Upper
By Skilled providers	Doctor	28(5.9)	3.8	8.0
	Health officer	21(4.4)	2.6	6.3
	Midwife/Nurse	188 (39.5)	35.1	43.9
	Unclassified skilled provider	11(2.3)	1.0	3.7
	Total	248(52.1)	47.6	56.6
By Non-skilled providers	HEW	28(5.9)	3.8	8.0
	TBA	4(0.8)	0.2	1.3
	Relatives	2(0.4)		
	Total	34(7.1)	4.8	9.5
Total assisted (skilled & Non skilled)		282(59.2)	54.8	63.7
No Assistance		194(40.8)	36.3	45.2

### Reasons for not seeking assistance during complications

**Figure 1:**
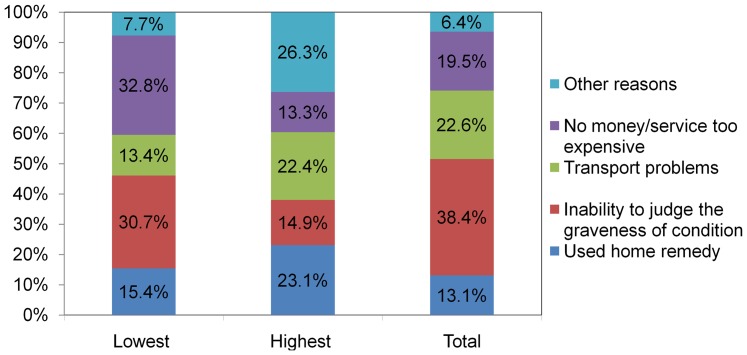
The main reasons for not seeking assistance at the time obstetric complications, North Gondar 2012.

Some of the women who reported complications did not seek services due to the following reasons: Inability to judge the graveness of condition (38.4%), distance/transport problems (22.6%), lack of money/cost considerations (19.5%), and use of traditional options at home (13.1%). The reasons varied with relative difference in wealth. The main reason for the lowest (poorest) quintile was lack of money/cost consideration (32.8%), followed by inability to judge the graveness of condition (30.7%), and use of home remedy (15.4%), whereas the main reasons for the wealthiest quintile were others, mainly quality of service (26.3%), use of home options (23.1%) and transport problems (22.4%) (Figure 1).

### Factors associated with seeking assistance at the time of obstetric complications

The multivariable analysis carried out using binary logistic regression indicated that three variables: the wealth index, having ANC by a skilled provider, and agreement of a woman to plan for possible complications were the factors found to be significantly associated with seeking assistance from a skilled provider. Wealthier women (who belonged to the second quintile) were 2.5 times (OR = 2.5, 95% CI; 1.3, 4.6) more likely to be assisted by a skilled provider at the time of obstetric complication than women in the poorest quintile. Women who had antenatal care by a skilled provider were about two times (OR = 1.7, 95% CI; 1.1, 2.7) more likely to seek assistance from a skilled provider during obstetric complication compared with women who did not have antenatal care. Similarly, mothers who agreed to plan for possible complications were 2.5 times (OR = 2.5, 1.6, 3.9) more likely to seek assistance from a skilled provider at the time of obstetric complication compared with women who did not agree (Table 5). The significant association observed during the bivariate analysis of some variables, like birth order and education of mothers and their husbands disappeared during the multivariable analysis.

**Table 5:** Multivariable analysis of factors associated with assistance by a skilled provider at the time of obstetric complications among women who gave birth within one year preceding the survey, North Gondar 2012.

Variables		n = 476	Assisted by a skilled provider		Adjusted OR	95.0% C.I.	
			Yes (%)	No (%)		lower	Upper
Number of births	1	87	62.1	37.9	1.0		
	2–3	186	46.8	53.2	0.5	0.3	1.1
	4–5	122	54.1	45.9	1.0	0.6	1.8
	6+	81	50.6	49.4	0.8	0.4	1.4
Mother's education	Illiterate	354	52.3	47.7	1.0		
	Primary	79	43.0	57.0	1.0	0.4	2.5
	Secondary & above	43	67.4	32.6	1.5	0.6	4.0
Husband's education	Illiterate	323	50.5	49.5	1.0		
	Primary	103	52.4	47.6	1.1	0.5	2.6
	Secondary & above	50	62.0	38.0	1.3	0.6	3.1
Wealth index	Lowest	98	36.7	63.3	1.0		
	Second	97	56.7	43.3	2.5*	1.3	4.6
	Middle	83	47.0	53.0	1.1	0.6	2.1
	Fourth	91	56.0	44.0	1.7	0.9	3.2
	Highest	107	62.6	37.4	1.2	0.6	2.1
Have ANC by a skilled provider	No	315	46.0	54.0	1.0		
	Yes	161	64.0	36.0	1.7*	1.1	2.7
Have agreement to plan for possible complications	No	115	33.9	66.1	1.0		
	Yes	361	57.9	42.1	2.5*	1.6	3.9

* Significant associations, P<0.05.

## Discussion

This study attempted to assess experiences related to obstetric complication and seeking assistance from a skilled provider among women who gave birth in the last 12 months prior to the study. The multivariable analysis showed that the agreement of a woman to plan for possible complications was one of the important factors found to be significantly associated with seeking assistance from a skilled provider. However, a significant proportion (40%) of women did not have any information about skilled maternal care or the importance of birth preparedness. The agreement to plan a delivery place and for possible complications was also much lower than the expectation.

In this study, having ANC by a skilled provider was significantly associated with seeking assistance from a skilled provider. The contribution of ANC to further maternal service utilizations, like delivery care, was observed in previous studies conducted in Ethiopia [16]. Its positive effect for timely care-seeking during obstetric complications was also observed in studies outside Ethiopia [17]. According to the standards of the World Health Organization, ‘All pregnant women should have a written plan for birth and for dealing with unexpected adverse events, such as complications or emergencies, and should discuss this plan with a skilled attendant at each antenatal assessment and at least one month prior to the expected date of birth’ [18]. However, only one-third of the women had ANC by a skilled provider.

The results of the study showed that nearly two-thirds of the mothers were able to mention at least one danger sign spontaneously though majority knew only bleeding. Bleeding was a common danger sign mentioned in another study in Adgrat, Ethiopia. However, fewer number of mothers (15.4%) spontaneously mentioned at least one key danger sign compared with our study [19]. This difference may be due to improvements in awareness by the current expansion of primary health care facilities and the Community Based Health Extension Program. In the Health Extension Program, the target of one health post and two health extension workers per kebele has been reached [20]. Globally, hemorrhage is the leading cause of maternal death. It is also the major known cause of maternal mortality in Sub-Saharan Africa, which accounts for about 34% of total deaths [21]. Retained placenta, prolonged labor (when the uterus fails to contract) and bleeding during pregnancy (due to low lying and early separation of placenta) are the common causes of hemorrhage. Awareness of danger signs or complications helps women to seek care timely.

About 28.5% of the women reported at least one kind of maternal morbidity perceived to be serious. Most women encountered these complications at the time of delivery and immediate postpartum period. This reflects the fact that most maternal deaths occur in the first week of the postpartum period, mainly in the first and second days after birth [1]. Excessive bleeding (named as ‘serakian’) was the common problem mentioned. Many studies reveal that hemorrhage is the most common type of obstetric complication and that it is the top direct obstetric cause of maternal death [1], [22]. According to further interviews of mothers and families experiencing complications, bleeding is associated with evil spirits. In their view, it is a killer unless managed immediately by the known traditional methods.

About half of the women who faced complications had sought assistance from skilled providers. Studies in other countries indicated higher percentage of care-seeking for maternal morbidities. For instance, in rural Haiti, 75% of pregnant women reported seeking care in the formal health sector for a pregnancy-related illness [23]. The differences may be due to the overall maternal service utilization pattern which is very low in Ethiopia [5]. Most of the assistances were by mid-level health care providers, nurses or midwives. This is because health centers are the common places for getting skilled providers, and such health professionals run most health centers.

This study revealed that some of the women who reported complications did not seek services due to reasons, like inability to judge the graveness of the condition, distance/transport problems, lack of money/cost considerations and use of traditional options at home. Inability to understand the severity of the problem was identified as one of the major reasons for delay or not seeking service at the time obstetric complications by different studies [24], [25].

In this study, the reasons for not seeking skilled providers at the time of obstetric complications differed according to wealth gradient. Lack of money/cost consideration, inability to judge the severity of the problem, and use of home remedy were the top reasons among the poorest women, whereas perceived quality of service and other reasons, such as use of home options and transport problems were the main reasons for the wealthiest women. In addition, women in the lowest wealth quintile were less likely to seek skilled providers at the time of complication compared with wealthier women in any other quintiles. The strong influence of the mothers' wealth status on the utilization of healthcare services during complications is consistent with findings of other studies conducted elsewhere. Many studies indicate that a woman belongs to the lowest economic status are highly associated with poor maternal service utilization, especially during delivery and emergency conditions [26], [27]. In some studies economic status emerges as a more crucial determinant of institutional care-seeking for child birth than access [28].

The other major reasons, identified by this study, including distance/transport problems, lack of money/cost considerations and use of traditional options at home were also observed in previous studies conducted in Ethiopia and abroad. A nationwide qualitative study in Ethiopia revealed that distance, cost and lack of support for the cultural practices around birth were major barriers for seeking maternal care. Seeking care for complications was considered only after traditional options, like local or herbal remedies and prayer were tried and proved unsuccessful [29]. According to the estimates of clients and data collectors, the average distance to reach a nearby basic obstetric care facilities (health centers) was 5.5±4 kilometers with a range of 1.5 to 15 kilometers. The mean travel time on foot was about 57.7±38.4 minutes, with a range of 10–120 minutes. In the case of some complications, like obstructed labor, women need to travel long distances to reach a hospital with comprehensive emergency obstetric care (that is only Gondar Hospital in the zone). Many districts like Janamora, Beyeda, Quara, Abderafi and Tselemt are 200 kilometer and beyond from the hospital. Cost of transport was the most important expenditure mentioned by women. When referred to the hospital, women need to pay up to Birr 1200 ($67) to rent a car. Women also mentioned expenses to buy supplies at health facilities. Currently, the Ministry of Health is distributing at least one ambulance to each district and avail free maternal services for poor women. However, the problem of transportation and service availability remain persistent challenges in the area.

Preferences for traditional options were related to perceptions and beliefs about the causes of complications. This study revealed that the majority of the mothers preferred to stay at home for the first 10 days, and only a few (15.2%) of the respondents supported the idea that leaving the home/home yard/in the first seven days after delivery is safe. This was identified as an important barrier to seek service for most of the complications, which usually occur during the postnatal period. According to the local tradition, postpartum complications and postpartum psychosis (in Amharic, ‘Menekat’) are associated with evil spirits with different names (‘tselay or tselay senay’, ‘tezewawari’, ‘telalafi’, ‘buda’). According to the explanations of respondents, families must look after the mother in her early postpartum period and never leave her all alone in the house. The community believes that a plant called “Demeketir” is one of the traditional remedies for stopping excessive bleeding at home. They believe that it can stop the bleeding when tied to the mother's hand, neck or waist, even at the time of cutting the plant.

### Limitations

The perception of symptoms of morbidity by women may vary compared with a trained personnel or when measured with certain instruments. Therefore, the reliability of self-reported complications based on a woman's recall may be limited compared to the results of medical examinations at health care facilities. Judging the severity of the illness is also a challenge. However, women's self-report will be the primary option for capturing maternal morbidity in places where women do not usually use skilled care providers or facilities for maternal services. The fact that women who died from obstetric complications were excluded from the study, might underestimate the results.

## Conclusions

This study showed that nearly half of the women who faced complications did not use skilled providers at the time of obstetric complications. The listed reasons and the multivariate analysis indicated that cognitive, geographic, economic and cultural barriers were involved for not using skilled maternal care at the time of obstetric complications.

Health behavior education on obstetric complications and local cultural beliefs is very important, especially for the poor, and for those who do not come for ANC. Geographic and economic access should be improved through strengthening community-based maternal services by health centers and should be carefully tailored to the local cultural beliefs. Moreover, ensuring comprehensive emergency obstetric care at rural hospitals and providing free maternal health care services are crucial steps to address the problem.
